# Quantitative and Qualitative Assessment of Yttrium-90 PET/CT Imaging

**DOI:** 10.1371/journal.pone.0110401

**Published:** 2014-11-04

**Authors:** Ali Asgar Attarwala, Flavia Molina-Duran, Karen-Anett Büsing, Stefan O. Schönberg, Dale L. Bailey, Kathy Willowson, Gerhard Glatting

**Affiliations:** 1 Medical Radiation Physics/Radiation Protection, Universitätsmedizin Mannheim, Medical Faculty Mannheim, Heidelberg University, Mannheim, Germany; 2 Institute of Clinical Radiology and Nuclear Medicine, Universitätsmedizin Mannheim, Medical Faculty Mannheim, Heidelberg University, Mannheim, Germany; 3 Department of Nuclear Medicine, Royal North Shore Hospital, Sydney, Australia; 4 Institute of Medical Physics, School of Physics, University of Sydney, Sydney, NSW, Australia; Stanford University School of Medicine, United States of America

## Abstract

Yttrium-90 is known to have a low positron emission decay of 32 ppm that may allow for personalized dosimetry of liver cancer therapy with ^90^Y labeled microspheres. The aim of this work was to image and quantify ^90^Y so that accurate predictions of the absorbed dose can be made. The measurements were performed within the QUEST study (University of Sydney, and Sirtex Medical, Australia). A NEMA IEC body phantom containing 6 fillable spheres (10–37 mm ∅) was used to measure the ^90^Y distribution with a Biograph mCT PET/CT (Siemens, Erlangen, Germany) with time-of-flight (TOF) acquisition. A sphere to background ratio of 8∶1, with a total ^90^Y activity of 3 GBq was used. Measurements were performed for one week (0, 3, 5 and 7 d). he acquisition protocol consisted of 30 min-2 bed positions and 120 min-single bed position. mages were reconstructed with 3D ordered subset expectation maximization (OSEM) and point spread function (PSF) for iteration numbers of 1–12 with 21 (TOF) and 24 (non-TOF) subsets and CT based attenuation and scatter correction. Convergence of algorithms and activity recovery was assessed based on regions-of-interest (ROI) analysis of the background (100 voxels), spheres (4 voxels) and the central low density insert (25 voxels). For the largest sphere, the recovery coefficient (RC) values for the 30 min –2-bed position, 30 min-single bed and 120 min-single bed were 1.12±0.20, 1.14±0.13, 0.97±0.07 respectively. For the smaller diameter spheres, the PSF algorithm with TOF and single bed acquisition provided a comparatively better activity recovery. Quantification of Y-90 using Biograph mCT PET/CT is possible with a reasonable accuracy, the limitations being the size of the lesion and the activity concentration present. At this stage, based on our study, it seems advantageous to use different protocols depending on the size of the lesion.

## Introduction

Liver cancer is a very frequent cancer, and liver metastases from tumors of other origin are a very common problem in oncology. Treatment options include external beam radiotherapy, chemotherapy or surgery. External beam radiotherapy (EBRT) can also affect normal liver tissue, and it can be very challenging or even impossible to surgically remove multiple liver lesions. [1], [2].

Recently, liver cancer treatment using ^90^Y in Selective Internal Radiation Therapy (SIRT) was established [2]–[10]. SIRT is based on microspheres containing radioactive ^90^Y, which are directly administered via super selective injection into selected branches of the hepatic artery, providing a highly localized dose to shrink the tumor before surgery or chemotherapy [11], [12]. Post treatment activity quantification, until recently was performed using the bremsstrahlung spectrum of ^90^Y captured with single photon emission computed tomography (SPECT) [13], [14]. However, the SPECT based method suffers from inaccuracies in quantification, ranging from 58% (37 mm sphere) to 75% (10 mm sphere) due to its low spatial resolution, photon scatter and collimator septal penetration [15].

Since the 1950′s it has been known that ^90^Y has minor positron decay [16], [17]. In 2004 Nickles et al. demonstrated the possibility of quantifying ^90^Y distribution using positron emission tomography (PET)[18]. The branching ratio was verified by Selwyn et al as 31.86+/−0.47×10^−6^ [19]. In 2010, Lhommel et al. performed a feasibility study to obtain the biodistribution of ^90^Y using time of flight (TOF) PET/CT and showed that this method provides higher accuracy for dose distribution assessments [20].

For dosimetry, quantitative imaging of the biokinetics of the radiolabelled substance is needed [21]. Hence, post administration activity quantification becomes challenging due to the low statistics of positron emission decay of ^90^Y (32 parts per million, ppm) combined with the high additional background from the bremsstrahlung and the limited resolution of the system [22].

There is a growing interest in investigating ^90^Y quantification, based on phantom and post radioembolization patient studies, to improve its accuracy and define a standard protocol for image acquisition and reconstruction [23], [24]. The quantitative accuracy of ^90^Y -PET/CT was recently investigated by Willowson et al. [25] in an experimental study with the IEC NEMA phantom and concluded that it has potential for deriving accurate dosimetric data. Carlier et al. demonstrated that spheres greater than 17 mm having an activity concentration of greater than 2 MBq/ml had up to 70% of the original activity recovered [26]. Influence of system linearity and parameter impact on ^90^Y dosimetry was investigated recently by Goedicke et al [27].

Here, we image and quantify ^90^Y using an IEC NEMA body phantom to investigate if accurate predictions of the absorbed dose can be made and thus if ^90^Y PET is suited for individualized dosimetry of SIRT. The measurements were performed at the occasion of the QUEST phantom study, a multi-center international study being run by The University of Sydney and Sirtex Medical, and the data was used to investigate sensitivity, calibration and quantification for different image reconstruction algorithms and acquisition times.

## Materials and Methods

### PET/CT

A Siemens Biograph 40 mCT PET/CT that has 4×4×20 mm^3^ lutetium oxyorthosilicate (LSO) based crystals arranged in blocks of 13×13 elements and 4 photomultiplier tubes per block was used. The investigated system consists of three detector rings; each of them made of 48 blocks containing 624 detector elements. The coincidence window was set to 4.5 ns. The axial and transaxial fields of view (FOVs) are 162 mm and 700 mm, respectively; the CT has a maximal transaxial FOV of 780 mm (“extended FOV”), the attenuation corrections of the reconstructed PET images were based on the CT data [28].

### Phantom

To simulate the activity distribution and uptake in a patient organ, the International Electrotechnical Commission (IEC) body phantom designed by the National Electricals Manufacturers Association (NEMA) organization was employed during the experiment. The phantom consists of six fillable spheres of different inner diameters (37 mm, 28 mm, 22 mm, 17 mm, 13 mm, and 10 mm). These spheres are attached via capillary tubes of 1.5 mm diameter opening to the top lid of the phantom. The respective measured volume of each sphere in ml is 26.52, 11.49, 5.57, 2.57, 1.15, and 0.52 [29].

A lung insert, which is a cylinder inserted in the center of the phantom body, has an inner diameter of 44.5 mm and a volume of 194 ml. The lung insert was filled with low-density polystyrene balls and water to simulate human lung density. Approximately 9.85 l of non-radioactive water was required for filling the phantom completely [29].

### Phantom Measurement

The phantom was prepared as in the QUEST study protocol [30]. ^90^Y activity was determined using the PTW dose calibrator (Freiburg i. Br., Baden-Württemberg, Germany) to be 3165 MBq. The difference between the activity measured in the dose calibrator and the calibration of the supplier was in close agreement (3168 MBq, decay corrected value to the time of measurement). The residual activity in the ^90^Y vial measured in the dose calibrator was 64 MBq.

The phantom was filled with this activity with a sphere to background ratio of ∼8∶1. Thus, the activity concentration in the spheres was 2380 kBq/ml and in the phantom background it was 304 kBq/ml.

PET/CT measurements were performed over a week at days 0, 3, 5 and 7. The phantom was positioned with the centers of the spheres aligned with the center of the acquisition field of view and in the anterior position i.e. the 17 mm and 37 mm spheres were along the x-axis. A 30-minute acquisition of 2-bed positions in static mode was performed. This was followed by a single bed acquisition of 120 min in list mode with the spheres positioned at the center of the FOV.

As ^90^Y was not available in the Biograph mCT PET acquisition isotope selection panel, ^22^Na (half-life 2.6 years) was used. Hence, by the end of the image acquisition week the system assumed a decay of 0.5% occurring for Na-22. The activity entered in the system before image acquisition was hence decay corrected according to the initial activity present on day 0 and the half-life of ^90^Y.

To determine the residual background counts in the FOV, in the absence of the phantom, a blank acquisition of 30 min with 2 bed positions was performed.

### Image reconstructions

Iteration numbers ranging from 1–12 with 21 (TOF) and 24 (non-TOF) subsets and Point Spread Function/PSF recovery and ordered subset expectation maximization (OSEM) reconstructions were used for all measurements. All reconstructions were performed with a zoom of 1, a matrix size of 400×400 with pixels dimension of 2.04 mm, no filtering (all-pass filter) or post reconstruction Gaussian filter of 5 mm full width at half maximum (FWHM). CT based attenuation and scatter correction were used in all reconstructions.

### Sensitivity and calibration assessments

Sensitivity for a PET system can be defined as its capacity to detect annihilation radiation given in cps/kBq and is calculated as the ratio of the total net trues (cps) to the total activity (kBq). Here, the sensitivity was calculated from the counts acquired from the 30 min acquisitions and the 120 min acquisition on each of the four days of imaging. The branching ratio of 32 ppm of positron emissions was taken into account.

The calibration factor of the system obtained for a ^18^F phantom was used to compare the administered and reconstructed activity concentration in a background region (25×25 pixels). Therefore, the ratio of reconstructed to administered activity concentration was calculated [31].

### ROI analyses

Comparison of the two acquisition modes was done based on the convergence of different algorithms and the calculation of the recovery coefficients. Convergence of the four algorithms was assessed based on regions-of-interest (ROIs) and analysis of the background (100 voxels) and lung insert (25 voxels) using the transverse section through the axial center of the phantom. The activity concentration recovery in the spheres was assessed using 4 voxels centered in all spheres and the recovery coefficients were calculated as the ratio of the mean activity measured in the ROI to the true activity present in the respective sphere. The signal-to-noise ratio (SNR) was calculated as the difference between the sphere and the background ROIs compared to the noise in the background ROI [32], [33].

Uncertainty in the sensitivity and calibration factor analyses was calculated using Gaussian error propagation.

## Results

### Sensitivity and calibration measurements

**Figure 1:**
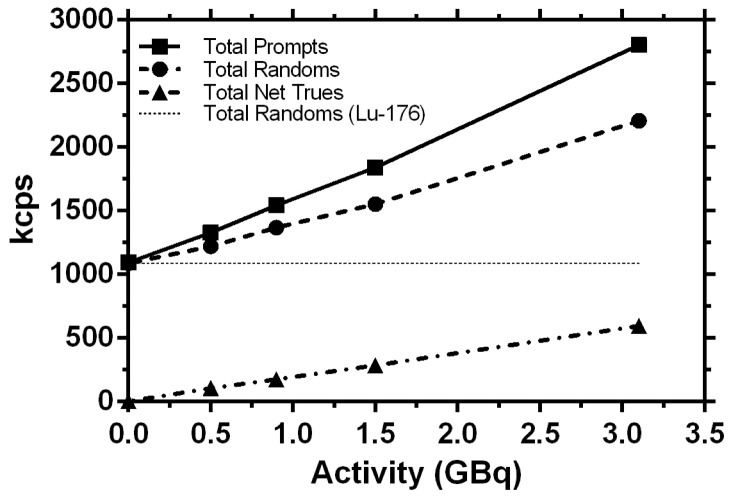
Counting statistics (kilocounts per second (kcps)) for the 30 min acquisition with 2 bed positions for Day 0, 3, 5 and 7 measurement time points during 1 week and an empty phantom measurement.

Total prompts, randoms and the net trues for the 30 min acquisition at the four time points are shown in Figure 1. Total prompts and random coincidences have an offset due to the contributions from the single photons from ^176^Lu. The net true coincidences fall along a straight line with a slope of (189.4±1.5) kcps/GBq, y-intercept of (6.8±2.5) kcps and R^2^ = 0.9998. The amount of inherent activity in the detectors due to the gamma emissions from ^176^Lu was estimated from the background trues to be approximately 2.77 MBq. The sensitivity of the system for ^90^Y measurements based on its positron emission was calculated to be (3.63±0.02) cps/kBq.

Accuracy of calibration was assessed based on the ratio of reconstructed to administered activity concentration *R* in a background region (25×25 pixels) and was calculated to be 0.93±0.02 for all measurements.

### Convergence of iterative image reconstruction

**Figure 2:**
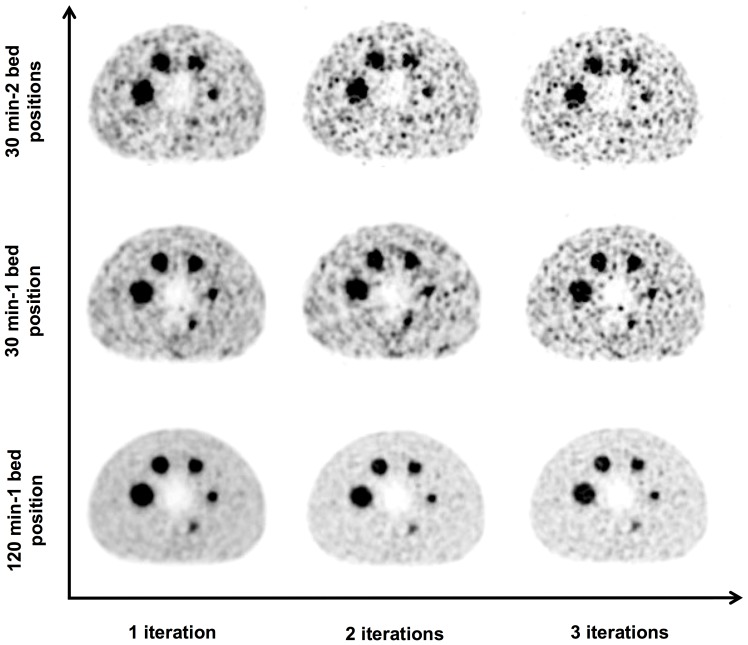
Transverse sections of the phantom reconstructed with 30 min-2 bed position, 30 min and 120 min single bed acquisitions and a matrix size of 400 and Gaussian filtering with FWHM of 5 mm for 21 subsets and 1–3 iterations of PSF TOF algorithms. Note that the smallest sphere is an empty sphere [40].

Figure 2 shows the effect of increasing iterations and different acquisition times on the reconstructed images. The visual quality of the images has a higher image contrast for the reconstructions made with a longer acquisition time. The 13 mm sphere (second smallest sphere) is easier to discern for the single bed 30 min and 120 min acquisitions in list mode as compared to 30 min static acquisition mode with 2 bed positions. Also the lung insert (cold region) is more clearly visible with the longer acquisition.

**Figure 3:**
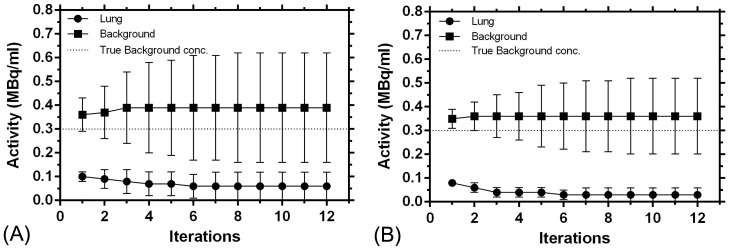
Convergence of the background (100 voxels) and lung insert (25 voxels) based on the transverse section through the axial center of the phantom, with the PSF TOF algorithm for the (A) 30 min-2 bed position acquisition and (B) 120 min-one bed position acquisition, reconstruction parameters: 1–12 iterations with 21 (TOF) subsets, 5 mm Gaussian filtering and an image matrix of 400.

**Figure 4:**
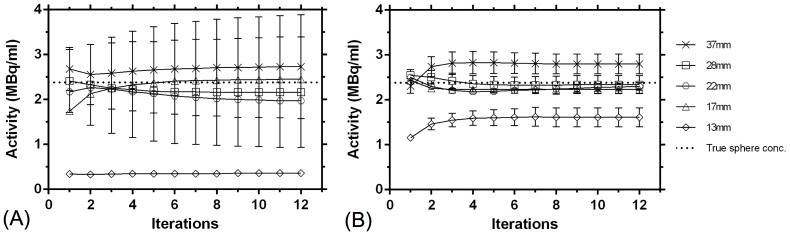
Convergence of the hot spheres (central 4 voxels in each sphere), with the PSF TOF algorithm for the (A) 30 min-2 bed position acquisition and (B) 120 min-one bed position acquisition, reconstruction parameters: 1–12 iterations with 21 (TOF) subsets, 5 mm Gaussian filtering and an image matrix of 400.

The figures 3 and 4 show the convergence of the images based on ROI analyses. For the PSF TOF algorithm and the 30 min acquisition, the background and lung reconstructed activity concentration change about 9% and 15%, respectively, from the first to the third iteration and 37% and 10%, respectively, from the first to the twelfth iteration (Fig.3A). For the 120 min acquisition, corresponding changes were 1% and 52%, and 2% and 61%, respectively (Fig.3B). The reconstructed activity concentration in the 37 mm and 28 mm spheres change about 3% and 7%, respectively, from the first to the third iteration and 2% and 10%, respectively, from the first to the twelfth iteration, for the 30 min acquisition (Fig.4A). For the 120 min acquisition, corresponding changes were 22% and 5%, and 22% and 8%, respectively (Fig.4B).

**Figure 5:**
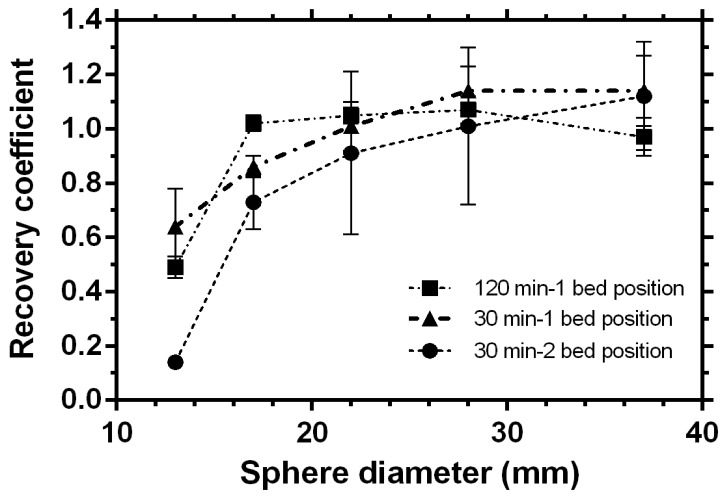
Recovery coefficients from day 0 acquisition for PSF TOF to demonstrate the effect of different acquisition times. Reconstruction parameters: 1 iteration, 21 (TOF) subsets, and image matrix of 400 and Gaussian filtering of 5 mm.

Figure 5 shows the change in recovery coefficients as a function of sphere diameters for images reconstructed with the Day 0 acquisition and with 1 iteration, 21 subsets and PSF TOF algorithm to see the effect of different durations of acquisition time. PSF correction with TOF reconstruction showed minimum standard deviations in recovery coefficients, as compared to other algorithms.

The recovery coefficients move towards one with increasing sphere diameters as expected. For the largest sphere of 37 mm diameter the best recovery achieved is within the standard deviations with the 30 min –2-bed position and 30 min and 120 min single bed position. The corresponding values are 1.12±0.20, 1.14±0.13, 0.97±0.07. For the spheres with diameters below 22 mm, the PSF algorithm with TOF provided better recovery compared to the OSEM algorithm and the non-TOF option. Also based on the acquisition times, the 30 min single bed acquisition provided a higher recovery compared to the 30 min 2 bed position acquisition.

**Figure 6:**
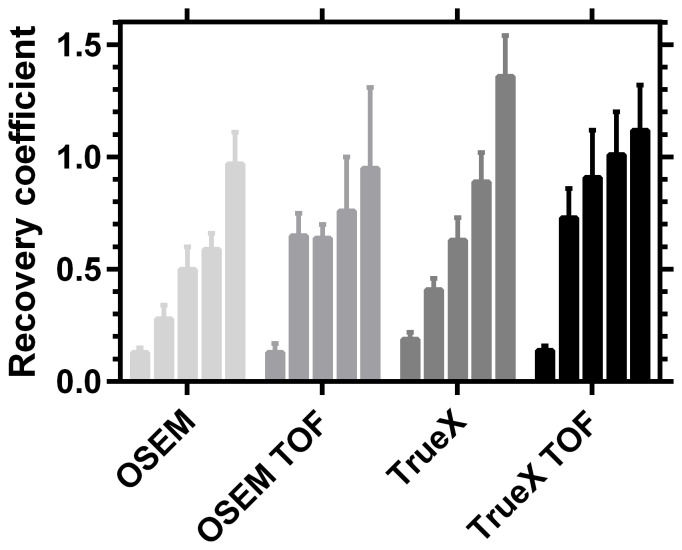
Recovery coefficients from day 0 acquisition for spheres with diamters (L to R: 13 mm, 17 mm, 22 mm, 28 mm and 37 mm) for OSEM TOF, PSF TOF, OSEM and PSF alogorithms respectively. Reconstruction parameters: 1 iteration, 24 and 21 (TOF) subsets, image matrix of 400 and Gaussian filtering of 5 mm.

Figure 6 shows a comparative analysis of the OSEM TOF, PSF TOF, OSEM and PSF algorithms based on the calculations of the recovery coefficients for the five spheres of different diameters and the 30 min- 2 bed position acquisition. The 17 mm sphere was the smallest sphere clearly discernible with 30 min 2 bed position and could be quantified with a RC of 0.73±0.14 using PSF TOF.

The best recovery is achieved with the 120 min single bed acquisition with all the four algorithms and the recovered activity for the 17 mm sphere for OSEM TOF, PSF TOF, OSEM and PSF algorithms were 0.95±0.06, 1.03±0.03, 0.60±0.05 and 0.80±0.03, respectively. The 13 mm sphere having a small size showed a larger influence of partial volume effects (PVE). The 13 mm sphere was discernible only with single bed acquisitions. The values of the recovery coefficients with 30 min 2 bed position acquisition for the phantom background on days 0, 3, 5 and 7 were 1.17±0.24, 0.87±0.40, 0.87±0.56 and 0.58±0.38, respectively.

## Discussion

The performance of Siemens Biograph 40 mCT PET/CT was investigated to quantitatively image a ^90^Y distribution and determine the effects of different acquisition times and reconstruction algorithms on the accuracy of quantification.

For quantification, system linearity is a prerequisite. The net trues in figure 1 fall along a straight line and show system linearity for an activity in the range of 0–3 GBq. This result is in good agreement with other previous studies [20], [25]. To investigate the differences in counts between ^18^F and ^90^Y, the number of total prompts, randoms and trues were recorded for a whole body patient acquisition of 30 min with approximately 179 MBq of FDG administered activity (decay corrected to the time of measurement). The ratio of randoms to trues was ∼0.6 for the ^18^F-FDG patient measurement and ∼3.7 for the ^90^Y phantom measurement. This indicates the large amount of singles that fall in the energy window from the gamma emission due to bremsstrahlung in the case of ^90^Y. The inherent activity of 2.77 MBq that was found to be present in the detectors due to ^176^Lu, was also investigated by Carlier et al. [26] and showed a similar result (2.6 MBq). Nevertheless, system linearity was preserved below 3 GBq.

From the sensitivities of 5.2 cps/kBq for the whole body patient mentioned above or 5.3 cps/kBq in the system specification of the Biograph mCT for ^18^F and 3.63±0.02 cps/kBq obtained for ^90^Y it can be seen that the system sensitivity for the ^90^Y measurement is lower by 32% than the standard value for ^18^F. A probable explanation for this decrease in sensitivity is a relatively large amount of high-energy ß^−^ falling in the energy window due to bremsstrahlung and also generally a tube phantom is used for standard sensitivity measurements. The calculated calibration factor of 0.93±0.02 indicates that the system dead time correction does not work appropriately for these additional bremsstrahlung photons.

Standard deviations (SD) in the background as well as the hot regions were higher for the 30 min 2 beds static acquisition compared with the 30 min single bed position in list mode acquisition. This is due to the positioning of the spheres in the center of the PET FOV, where the PET detectors offer maximum spatial resolution and also because of the better statistics with the single bed position acquisition (total net trues for the 30 min-2 bed position and the 30 min single bed position acquisitions were 595 kilocounts and 675 kilocounts respectively). For the 30 min static acquisition, reconstructed with OSEM TOF, PSF TOF, OSEM and PSF, the corresponding SD for background were 42%, 20%, 29% and 18% and for the 120 min single bed acquisition 13%, 10%, 9% and 8% respectively. The standard deviations also increase with an increase in the number of iterations (Fig. 4). This higher inhomogeneity in the background for all the acquisitions with increasing effective iterations can also be visually observed in figure 2.

Considering the fact that lowest standard deviations were shown in reconstructions having 1 iteration with 24 (non-TOF) and 21 (TOF) subsets (this was also recommended by the QUEST imaging protocol), most of the results assessed were reconstructed with 1 iteration. Although, keeping in mind that the algorithms converged at 3 iterations, all evaluations were done with both 1 and 3 iterations. Increasing the number of iterations from 1 to 3 showed a small improvement in quantification for all the spheres when reconstructed with PSF and PSF TOF. Although this may just be an effect of lower signal to noise with an increase in the number of iterations as seen in figure 2.

Ideally the lung insert should demonstrate no activity concentration since it is a cold region with zero injected activity. However, as seen from Fig. 3 the lung insert shows an activity concentration of 96±20 kBq/ml and 84±15 kBq/ml for the 30 min 2 bed position and the 120 min single bed acquisition respectively. This may be due to inadequate scatter correction performed by the system and maybe also a possible reason for the overestimation in the quantification of the background.

The assessment of the background recovery for days 0, 3, 5 and 7 respectively gave a measure of the recovery, independent of the influence of PVE and sphere diameter (lesion size). The comparatively small amount of activity concentration of 395 kBq/ml present in the spheres on D7 had a RC of 1.46±0.42 for the large sphere (37 mm) with PSF-TOF. This overestimation is probably a consequence of the spurious high pixel values due to the noisy reconstructions, as the sphere also showed a very poor SNR (2.3±1.5) on day 7.

For the smaller sphere sizes, the recovery coefficient is higher for single bed position acquisition. This was because of the higher sensitivity at the center of the axial FOV present for the single bed position, consequently leading to a higher number of net trues acquired.

PVE is a consequence of the limited resolution of the system affecting adversely activity quantification in small regions [25], [31], [33], [34]. Hence, for the smaller lesions PVE corrections with the calculated recovery coefficients becomes of critical importance and for a smaller amount of activity present on day 7 (even for the large 37 mm sphere), the influence of residual background activity due to LSO became very prominent and corrections for these two effects is of critical importance. Another factor that may influence the accuracy of quantification in a clinical setting is the PVE in small lesions in an elastic organ like liver. Since this was a phantom based study, the corrections for liver movement were not investigated here. However, methods like respiratory triggering, computational modeling approaches and PET data-driven respiratory gating and amplitude gating are already being investigated for organ motion correction in PET imaging [35] [36] [37] [38] [39].

Although for the large sphere, 30 min 2-bed, 30 min and 120 min single bed acquisitions provided similar results, a clear advantage of using PSF correction with TOF and a longer acquisition for activity recovery for smaller spheres is demonstrated in Fig. 5. However, in a real clinical situation involving a patient, a trade-off has to be made between image quality and the acquisition time and it would not be practical to scan the patient for more than 30 min.

For dosimetry, quantitative data are required and thus optimization of reproducibility of the used reconstruction algorithm is an important factor in accurate quantification [41]. One limitation of this study was that the reproducibility of the algorithms was not tested for small and bigger lesions in case of lower uptake values. However, this needs to be investigated and optimized in the future.

Size of the lesion and the amount of activity concentration were two important parameters to be considered during assessment of activity quantification. It was seen that although ^90^Y imaging is possible for large lesions and a high activity concentration, PVE, pixel size, low count statistics and intrinsic background activity from LSO influence the quantification of smaller lesions and smaller activity concentrations. Also it needs to be kept in mind that during this work a fixed ROI approach with a sample of 4 pixels was used. This 2×2 fixed ROI was placed at the center of the spheres to evade the effect of spill-in from the background. Although this method eliminates operator bias, it can be influenced by the sphere size or non-uniform activity distribution and in addition, a small sample of 4 pixels is susceptible to higher noise.

## Conclusion

Quantification of ^90^Y using Biograph mCT PET/CT is possible with a reasonable accuracy even with its very low branching ratio, the limitations being the size of the lesion and the amount of activity concentration present. At this stage, based on our study, it seemed advantageous to use different protocols depending on the size of the lesion. For large lesions, a protocol with 30 min 2-bed positions, 30 min and 120 min single bed acquisitions using 1 iteration seemed to give similar results, and with smaller lesions single bed acquisitions showed better quantification. The combination of PSF algorithm with TOF was able to produce the best results.

A matter of further investigation would be to test that the results using these protocols are repeatable with acceptable deviations and reproducible with different PET/CT imaging systems.

## Supporting Information

File S1 Recovery coefficients and the corresponding recovered activity concentrations for 30 min-2 bed position acquisition and 120 min-one bed position acquisition for OSEM TOF, PSF TOF, OSEM and PSF alogorithms with iterations 1–2, 24 and 21 (TOF) subsets, image matrix of 400 and Gaussian filtering of 5 mm along with the sensitivity analyses for each measurement day (days 0, 3, 5 and 7) based on the acquired prompts, net trues and randoms.
(XLSX)
