# Correction: Quantitative and Qualitative Assessment of Yttrium-90 PET/CT Imaging

**DOI:** 10.1371/journal.pone.0118423

**Published:** 2015-02-26

**Authors:** The PLOS ONE Staff

In the third sentence under the subheading “Sensitivity and calibration measurements” of Results, Fig. 1, and the legend for Fig. 1, the abbreviation for kilocounts is incorrect. Please view the corrected Fig. 1 below.

**Fig 1:**
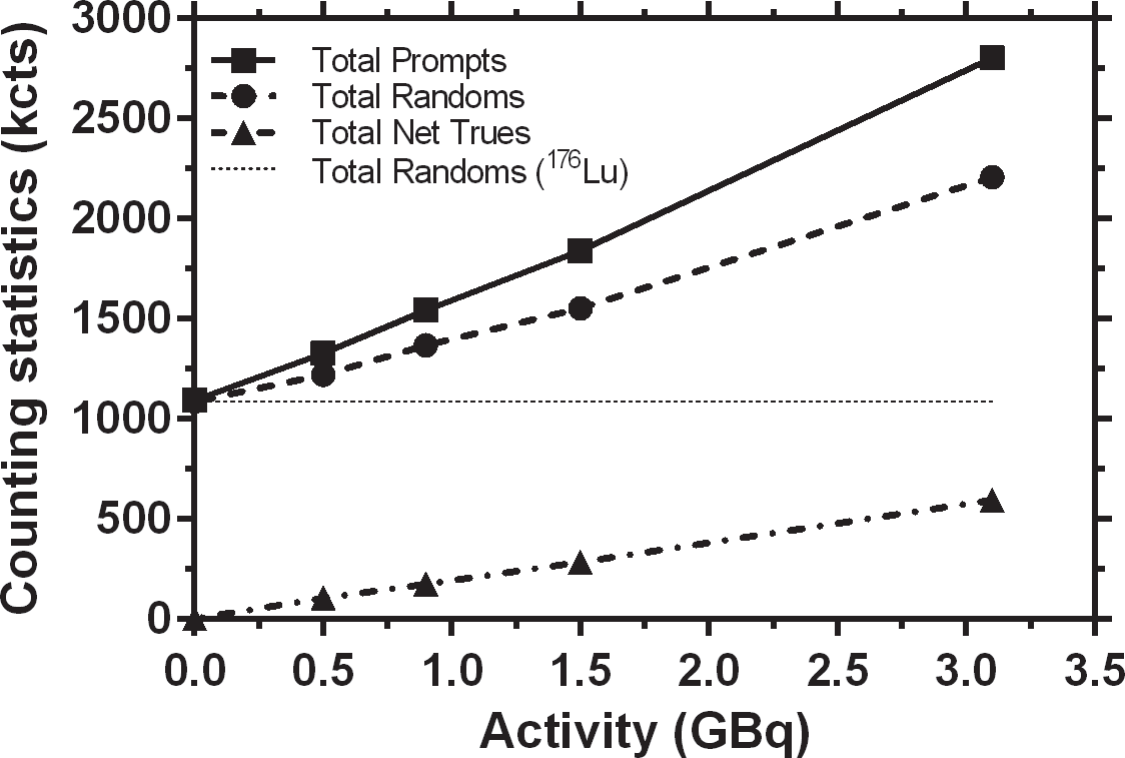
Counting statistics (kcts) for the 30 min acquisition with 2 bed positions for Day 0, 3, 5 and 7 measurement time points during 1 week and an empty phantom measurement.

The corrected third sentence under the subheading “Sensitivity and calibration measurements” of Results is: “The net true coincidences fall along a straight line with a slope of (189.4 ± 1.5) kcts/GBq, y-intercept of (6.8 ± 2.5) kcts and R2 = 0.9998.”

The complete, corrected Fig. 1 legend is: “Counting statistics (kcts) for the 30 min acquisition with 2 bed positions for Day 0, 3, 5 and 7 measurement time points during 1 week and an empty phantom measurement.”

## References

[1] Attarwala AA, Molina-Duran F, Büsing K-A, Schönberg SO, Bailey DL, Willowson K, et al (2014) Quantitative and Qualitative Assessment of Yttrium-90 PET/CT Imaging. PLoS ONE 9(11): e110401 doi:10.1371/journal.pone.0110401 25369020 10.1371/journal.pone.0110401 PMC4219690

